# The use of radiolucent pedicle fixation in degenerative lumbar spine surgery

**DOI:** 10.1093/jscr/rjab595

**Published:** 2022-01-21

**Authors:** Nathan Xie, Sihyong J Kim, Ralph J Mobbs, Rajesh Reddy

## Abstract

Radiolucent pedicle screw fixation has become popularized in the field of oncological spine surgery owing to its ability to provide superior post-operative planning for adjuvant radiotherapy and radiological monitoring of tumour progression. We present the case of a 45-year-old female with degenerative spine pathology who underwent L4/5 and L5/S1 posterior lumbar interbody fusion with carbon fibre reinforced-polyetheretherketone pedicle screw fixation. The authors highlight the potential advantages of radiolucent pedicle fixation, which may translate into the degenerative spine surgery domain.

## INTRODUCTION

Pedicle fixation for spinal stabilization has been a fundamental element of spinal fusion since its introduction in the 1960s [1]. While the technique itself has remained relatively similar over time, the screw materials have evolved significantly with ongoing research—from stainless steel to titanium alloy, and more recently, carbon fibre reinforced-polyetheretherketone (CFR-PEEK). CFR-PEEK pedicle screws have become recently popularized in the field of oncological spine surgery, where their radiolucent properties allow for superior post-operative planning for adjuvant radiotherapy and radiological monitoring of tumour progression [2, 3].

Employing CFR-PEEK screws in degenerative spine surgery may also carry similar benefits. Currently, titanium alloy screws remain the standard of care, providing high bioactivity and flexibility without loss of tensile strength, resulting in improved bone growth and mechanical fixation when compared to its predecessors [4, 5]. However, the major drawback of these screws lie in their metallic-induced artefacts on magnetic resonance imaging (MRI), which result in anatomical distortion and loss of signal, hampering the ability to radiologically assess post-operative progress, even with the aid of metal artefact reduction techniques [2, 6–8]. CFR-PEEK screws are less subject to these image-degrading qualities, given their lower inherent density and lack of metallic components [8]. As it is often soft tissue structures that must be visualized to determine the source of neural impingement, post-operative assessment in patients who experience recurrent symptoms would be greatly aided by the ability to accurately interpret computed tomography (CT) and MRI scans without significant image distortion due to artefact.

We therefore present the case of a 45-year-old female who underwent a L4/5 and L5/S1 posterior lumbar interbody fusion (PLIF) [9] with CFR-PEEK pedicle screw fixation for lower limb radiculopathy secondary to isthmic spondylolisthesis and spondylosis. This patient required frequent post-operative imaging for ongoing symptomatology, illustrating how CFR-PEEK screws can be particularly useful in degenerative spine surgery.

## CASE REPORT

**Figure 1:**
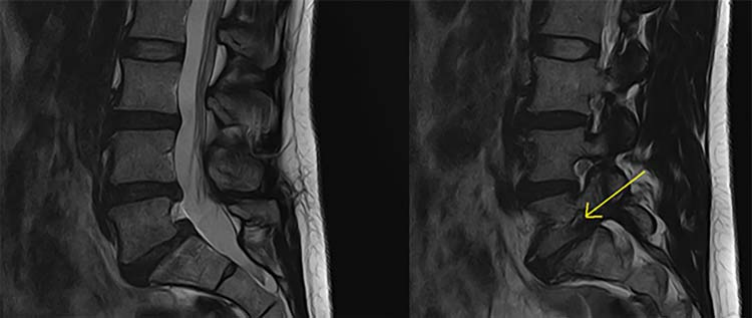
Preoperative MRI lumbar spine (mid- and para-sagittal slices of T2 weighted sequence) demonstrating posterior bulging of the L4/5 and L5/S1 discs in conjunction with a Grade 1 spondylolisthesis of L5 on S1 resulting in significant right-sided foraminal stenosis at L5/S1 (indicated by yellow arrow).

This 45-year-old female presented with low back pain and right-sided L5 radiculopathy involving pain and sensory symptoms extending into the leg. Her symptoms began following an acute event involving a bend, twist and lift injury. She trialled conservative measures, including analgesia, physiotherapy and perineural injections, over a period of 6 months, with persistent symptoms. Imaging revealed bilateral L5 pars defects and a Grade 1 spondylolisthesis resulting in foraminal stenosis with bilateral compression of the exiting right L5 nerve roots. In addition, degenerative disc disease was present at both L4/5 and L5/S1 with contact of the right L5 nerve root in both the right L4/5 lateral recess as well as within the right L5/S1 foramen (Fig. 1). A single photon emission CT bone scan was additionally performed, demonstrating significant uptake in the L4/5 and L5/S1 facet joints. Based on these findings, L4/5 and L5/S1 PLIF were recommended. The patient proceeded to surgery with CFR-PEEK pedicle screws (CarboFix, TelAviv, Israel) and PEEK interbody cages (Evolution Spine, Sydney, Australia) inserted at both levels. Operative time was 3.5 hours, with 240 ml of blood loss and no intraoperative complications.

**Figure 2:**
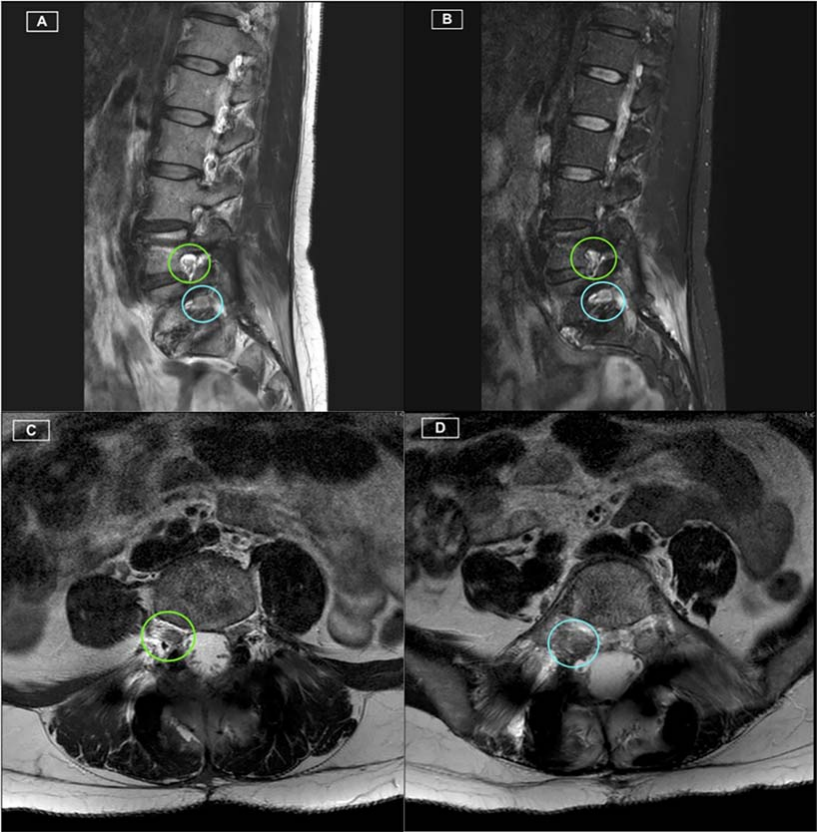
Postoperative MRI Lumbar spine. **(A)** Right-sided parasagittal slice of T2 Sequence MRI. Right L4/5 foramen (superior) and Right L5/S1 foramen (inferior) have been highlighted, with excellent definition of the Right L4 and L5 nerve roots. **(B)** Right-sided parasagittal slice of Short Tau Inversion Recovery (STIR) Sequence MRI. Exiting L5 nerve root is well visualised, with evident perineural thickening. **(C)** Axial slice of T2 Sequence MRI at the level of the L4/5 Foramen. The Right L4 nerve root is identified with excellent visualisation. **(D)** Axial slice of T2 Sequence MRI at the level of the L5/S1 Foramen. The Right L5 nerve root is identified with excellent visualisation.

**Figure 3:**
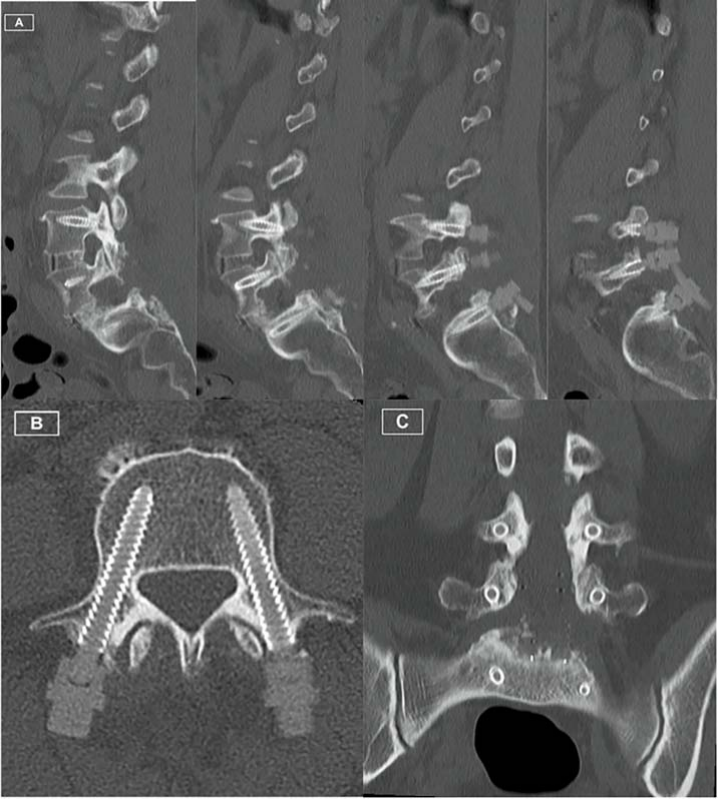
Postoperative CT Lumbar spine. **(A)** Sequence of sagittal slices demonstrating excellent definition of screw trajectory. **(B)** Axial slice through L4 pedicles demonstrating definition of screws and distinction from bony cortex. **(C)** Coronal slice demonstrating screws at all levels with no peri-structural artifact.

The patient recovered well, with resolution of her lower limb radiculopathy. Ongoing low back pain was managed by a multidisciplinary pain team. At 6 months post-operative, the patient experienced some recurrence of right leg pain. CT revealed excellent hardware position with no pseudarthrosis. MRI demonstrated adequate decompression of the descending and exiting L5 nerve roots, with minor perineural thickening within the right L5/S1 foramen. This patient was managed conservatively for her pain, given the lack of radiological pathology identified on CT and MRI. Figures 1–3 highlight the low artefact presence on both post-operative MRI and CT, allowing for optimal assessment of bony and neurological structures.

## DISCUSSION

Our case highlights how CFR-PEEK screw usage in degenerative spine cases can be advantageous for post-operative radiological assessment. Currently, there are limited data in the literature in this context, with only a single case series (*N* = 20) [8]. In this series, Fleege *et al.* were able to demonstrate a statistically significant improvement in the radiological assessment of surrounding anatomy when using CFR-PEEK screws (Icotec AG system) as opposed to titanium alloy screws (Expedium Spine System—DePuy Synthes Spine Inc). In particular, using a classification of radiological accessibility piloted in the study, the radiological accessibility of neuroforamina as well as the central canal in cases using CFR-PEEK screws were overwhelmingly rated as ‘very good’ (i.e. almost no artefact affecting the considered structure with pathologies clearly visible), whereas cases with titanium alloy screws were mostly rated as ‘fair’ (i.e. scattering effects of artefact resulting in only moderate accessibility of the considered structure). The study concluded that CFR-PEEK screws significantly improve the radiologically accessibility of anatomical structures following fusion in degenerative spine surgery, offering a diagnostic benefit in the detection of potential complications. These findings mirror those found in similar studies in the field of oncological spine surgery [2].

Given that CFR-PEEK screws are less dense than those constructed of titanium alloy, a potential downside may lie in the biomechanical strength of the fusion construct [10]. However, various studies have demonstrated that CFR-PEEK screws show comparable biomechanical strength to titanium alloy screws and potentially even superior fusion-promoting qualities, given their elastic properties that mimic the spine’s natural physiological response to applied forces [10–13]. These properties have allowed CFR-PEEK screws to achieve minimal screw loosening rates in controlled testing environments over multiple load cycles, which were found to be even lower than that of titanium alloy screws [10]. The available evidence therefore suggests that there is no biomechanical disadvantage when comparing CFR-PEEK to titanium alloy. However, the concept of revision surgery, or extension of the fusion construct to adjacent segments, is at this stage unreported in the literature. With the potential increased usage of CFR-PEEK screws for degenerative pathologies, these issues will need to be addressed in further studies.

## CONCLUSIONS

Our case highlights the advantages of CFR-PEEK screws in degenerative spine surgery. Given the high rates of symptom recurrence in this field, radiolucent screws allowing for improved imaging to assess implicated structures may offer a significant diagnostic benefit, with no currently reported biomechanical downsides. Further *in vivo* studies comparing CFR-PEEK to titanium alloy screws in degenerative spine cases would be recommended to confirm clinical benefit and to determine long-term outcomes.
